# Larvicidal and adulticidal effects of some Egyptian oils against *Culex pipiens*

**DOI:** 10.1038/s41598-022-08223-y

**Published:** 2022-03-15

**Authors:** Mohamed M. Baz, Abdelfattah Selim, Ibrahim Taha Radwan, Abeer Mousa Alkhaibari, Hanem F. Khater

**Affiliations:** 1 Department of Entomology, Faculty of Science, Benha University, Benha, 13518 Egypt; 2 Department of Animal Medicine (Infectious Diseases), Faculty of Veterinary Medicine, Benha University, Toukh, 13736 Egypt; 3 Supplementary General Sciences Department, Faculty of Oral and Dental Medicine, Future University in Egypt, P.O. Box 11835, Cairo, Egypt; 4 Department of Biology, Faculty of Science, University of Tabuk, Tabuk, 71491 Kingdom of Saudi Arabia; 5 Department of Parasitology, Faculty of Veterinary Medicine, Benha University, Toukh, 13736 Egypt

**Keywords:** Developmental biology, Ecology

## Abstract

Mosquitoes and mosquito-borne diseases represent an increasing global challenge. Plant extract and/or oils could serve as alternatives to synthetic insecticides. The larvicidal effects of 32 oils (1000 ppm) were screened against the early 4th larvae of *Culex pipiens* and the best oils were evaluated against adults and analyzed by gas chromatography-mass spectrometry (GC mass) and HPLC. All oils had larvicidal activity (60.0–100%, 48 h Post-treatment, and their Lethal time 50 (LT_50_) values ranged from 9.67 (*Thymus vulgaris*) to 37.64 h (*Sesamum indicum*). Oils were classified as a highly effective group (95–100% mortalities), including *Allium sativum, Anethum graveolens, Camellia sinensis, Foeniculum vulgare, Nigella sativa, Salvia officinalis, T. vulgaris,* and *Viola odorata.* The moderately effective group (81–92% mortalities) included *Boswellia serrata, Cuminum cyminum, Curcuma aromatic, Allium sativum, Melaleuca alternifolia, Piper nigrum,* and *Simmondsia chinensis*. The least effective ones were *C. sativus* and *S. indicum. Viola odorata, Anethum graveolens, T. vulgaris,* and *N. sativa* provide 100% adult mortalities PT with 10, 25, 20, and 25%. The mortality percentages of the adults subjected to 10% of oils (H group) were 48.89%, 88.39%, 63.94%, 51.54%, 92.96%, 44.44%, 72.22%, and 100% for *A. sativum, An. graveolens, C. sinensis, F. vulgare, N. sativa, S. officinalis, T. vulgaris,* and *V. odorata,* respectively. *Camellia sinensis* and *F. vulgare* were the most potent larvicides whereas *V. odorata, T. vulgaris, An. graveolens* and *N. sativa* were the best adulticides and they could be used for integrated mosquito control.

## Introduction

Mosquitoes are an ancient nuisance pest and mosquito-borne diseases represent an increasing global health challenge, threatening over 40% of the world’s population and it is expected that almost half of the world’s population will be at risk of arbovirus transmission by 2050^1^. *Culex pipiens* (Diptera: *Culicidae*) is widely distributed, transmitting dreadful diseases leading to severe morbidity and sometimes mortality to humans and animals^2–5^.

Vector control is the primary method for reducing public concerns about mosquito-borne diseases^6–11^. Controlling adults and larvae through repellents and insecticides^12,13^, are the most effective approach for reducing mosquito bites. Using synthetic insecticides led to insecticide resistance, environmental pollution, and health hazards to human health and non-target organisms.

Searching for eco-friendly alternatives in botanicals such as essential oils (EOs) is a curtail need. EOs are volatile components found in many plant families like Asteraceae, Rutaceae, Myrtaceae, Lauraceae, Lamiaceae, Apiaceae, Piperaceae, Poaceae, Zingiberaceae, and Cupressaceae^14^. EOs contain complicated mixtures of products as phenols, sesquiterpenes, and monoterpenes^15^.

EOs have antibacterial, antiviral, and antifungal activities. They also possess insecticidal effect interfering with insects' physiological, metabolic, behavioral, and biochemical functions through inhalation, ingestion, or skin absorption of EOs inducing a neurotoxic action^16^. EOs act as adulticides, larvicides, deterrents, and repellents. They are less toxic, biodegradable, and overcome insecticidal resistance^15,17,18^.

EOs have higher popularity with organic growers and environmentally conscious consumers and suitability for urban areas, homes, and other sensitive areas.

The role of EOs in mosquito control has been discussed^15,19^. This study aimed to screen and evaluate the lethal time values of the larvicidal effects of thirty-two oils and evaluate the adulticidal effect and phytochemical analyses of the most effective ones against *Cx. pipiens.*

## Materials and methods

### Plant oils

Thirty- two oils were purchased from EL CAPTAIN Company for extracting natural oils, plants, and cosmetics "Cap Pharm," El Obor, Cairo, Egypt and Harraz for Food Industry & Natrual products, Cairo, Egypt (Table 1).

**Table 1:** Plants species screened (oil No = 32) used for larvicidal activity.

No.	Oil name	Plant oils		
		Order	Family	English name
1	*Allium sativum*^a^	Asparagales	Amaryllidaceae	Garlic
2	*Anethum graveolens*^a^	Apiales	Apiaceae	Dill
3	*Argania spinosa*^b^	Ericales	Sapotaceae	Argan
4	*Boswellia serrata R.*^a^	Sapindales	Burseraceae	Olibanum
5	*Brassica carinata*^a^	Brassicales	Brassicaceae	Mustard
6	*Camellia sinensis*^a^	Ericales	Theaceae	Green Tea
7	*Cedrus libani A*^a^	Pinales	Pinaceae	Cedar wood
8	*Citrullus colocynthis L*^b^	Cucurbitales	Cucurbitaceae	Bitter apple
9	*Crocus sativus L.*^a^	Asparagales	Iridaceae	Saffron crocus
10	*Cucurbita maxima D.*^a^	Cucurbitales	Cucurbitaceae	Pumpkin
11	*Cuminum cyminum L*^a^	Apiales	Apiaceae	Cumin
12	*Cupressus sempervirens*^b^	Pinales	Cupressaceae	Italian cypress
13	*Curcuma aromatica S.*^a^	Zingiberales	Zingiberaceae	Curcuma
14	*Curcuma longa L.*^a^	Zingiberales	Zingiberaceae	Common turmeric
15	*Foeniculum vulgare M.*^a^	Apiales	Apiaceae	Sweet fennel
16	*Gadus morhua*^a^	Gadiformes	Gadidae	Cod Liver
17	*Lepidium sativum L.*^a^	Brassicales	Brassicaceae	Garden pepperwort
18	*Linum usitatissimum L.*^a^	Malpighiales	Linaceae	Common flax
19	*Melaleuca alternifolia*^a^	Myrtales	Myrtaceae	Tea tree
20	*Nigella sativa*^a^	Ranunculales	Ranunculaceae	Black cumin
21	*Panax ginseng*^a^	Apiales	Araliaceae	Chinese ginseng
22	*Piper nigrum L.*^a^	Piperales	Piperaceae	Black pepper
23	*Prunus dulcis*^b^	Rosales	Rosaceae	Almond
24	*Ruta chalepensis L.*^a^	Sapindales	Rutaceae	Rues
25	*Salvia officinalis L.*^a^	Lamiales	Lamiaceae	Sage
26	*Sesamum indicum*^a^	Lamiales	Pedaliaceae	Sesame
27	*Simmondsia chinensis*^b^	Caryophyllales	Simmondsiaceae	Jojoba
28	*Syzygium aromaticum L*	Myrtales	Myrtaceae	Clove
29	*Tilia americana L.*^a^	Malvales	Malvales	Tilia
30	*Thymus vulgaris L*	Lamiales	Lamiaceae	Garden
31	*Viola odorata L.*^a^	Malpighiales	Violaceae	Sweet violet
32	*Zingiber officinale*^a^	Zingiberales	Zingiberaceae	Ginger

^a^Plant oils purchased from EL CAPTAIN company for extracting natural oils, plants and cosmetics “Cap Pharm”.

^b^Plant oils purchased from Harraz for Food Industry & Natural products.

### Culex pipiens

*Culex pipiens* (anautogenous strain) was provided from the colony reared at the Department of Entomology, Faculty of Science, Benha University, Egypt, and maintained at 27 ± 2 °C, 75–85% RH and 14: 10 h (L/D) photoperiod.

### Larvicidal efficacy

Thirty-two oils were screened for their larvicidal efficacy^20^ against the early fourth instar larvae, *Cx. pipiens.* Oils were added to a solvent (emulsifier) consisting of dechlorinated water plus 1.0 mL 0.5% Tween-20, through a shaker plate to yield a homogenous solution. Oils were added to a solvent consisting of dechlorinated water plus 5% tween 20. For each oil, twenty larvae were placed in a 500 mL glass beaker containing 250 mL of 1000 ppm. The experiment and the control group, treated with the solvent only, were replicated three times. Larval mortalities were recorded 0.5, 2, 8, 24, and 48 h post-treatment (PT).

### Adulticidal efficacy

Susceptibility tests for adult mosquitoes were performed for the promising larvicidal oils through the CDC bottle bioassays^21^ with modifications. For each concentration, three bottles were coated. Several concentrations for each oil were prepared using pure ethanol as a solvent. The bottles were coated with the desired concentrations and left overnight at 27** ± **2 °C for solvent evaporation.

Adult mosquitoes (15–10, aged 3–4 days) fed on 10% sucrose solution were released to each bottle using a hand aspirator. The exposure time was set to 30 min. The mosquitoes were removed from the bottles. Mosquito groups were added to separate transparent paper cups (10 × 9 × 6 cm) having 10% sucrose solution and mortalities were checked after 24 h. Three replicates were made for each concentration.

### GC/MS analysis

A Thermo Scientific Trace GC Ultra/ISQ Single Quadrupole MS, TG-5MS fused silica capillary column was used for the GC/MS study (0.1 mm, 0.251 mm and 30 m film thickness). An electron ionisation device with a 70 eV ionisation energy was employed for GC/MS detection. At a constant flow rate of 1 mL/min, helium gas was used as the carrier gas. Temperatures were established at 280 °C for the injector and MS transfer line. The oven temperature was set at 50 °C (hold for 2 min), then increased to 150 °C at a rate of 7 °C per minute, then to 270 °C at a rate of 5 °C per minute (hold for 2 min), and finally to 310 °C at a rate of 3.5 °C per minute (hold 10 min). A percent relative peak area was used to explore the quantification of all of the discovered components. The chemicals were tentatively identified by comparing their respective retention times and mass spectra to those of the NIST, WILLY library data from the GC/MS instrument. The identification was done using mass spectra and a computer search of user-generated reference libraries. To check peak homogeneity, single-ion chromatographic reconstruction was used. When identical spectra could not be identified, only the structural type of the relevant component was provided based on its mass spectral fragmentation. When possible, reference compounds were co-chromatographed to confirm GC retention durations^22^.

### Data analysis

Data were analyzed through one-way analysis of variance (ANOVA), Duncan’s multiple range tests, and Probit analysis for calculating the lethal concentration (LC) and lethal time (LT) values using the computer program PASW Statistics 2009 (SPSS version 22). The relative efficacies (RE) were calculated^18^ according to the following formula:

$$\begin{aligned} & {\text{RE}}\;{\text{for}}\;{\text{LC}} = {\text{LC}}_{50} \left( {{\text{LC}}_{90} \;{\text{or}}\;{\text{LC}}_{99} } \right)\;{\text{for}}\;{\text{refernce}}\;{\text{oil}}/{\text{LC}}_{50} \left( {{\text{LC}}_{90} \;{\text{or}}\;{\text{LC}}_{99} } \right)\;{\text{for}}\;{\text{EO}}. \\ & {\text{RE}}\;{\text{for}}\;{\text{LT}} = {\text{LT}}_{50} \left( {{\text{LT}}_{90} \;{\text{or}}\;{\text{LT}}_{99} } \right)\;{\text{ for}}\;{\text{reference}}\;{\text{oil}}/{\text{LT}}_{50} \left( {{\text{LT}}_{90} \;{\text{or}}\;{\text{LT}}_{99} } \right)\;{\text{for}}\;{\text{EO}}. \\ \end{aligned}$$

Non-parametric, Kruskal–Wallis test was performed to compare the mean differences of more than two groups followed by the Mann–Whitney test to compare the mean differences between the effective oil groups.

## Results

The larvicidal effect of 32 oils was screened against the early 4th larvae, *Cx. pipiens*. The results showed that all plant oils had larvicidal activity (60.0–100%, 48 h PT) and their Lethal time 50 (LT_50_) values ranged from 9.67 (*Thymus vulgaris*) to 37.64 h (*Sesamum indicum*), Tables 2 and 3.

**Table 2:** Larval mortality (%) of plant oils used at 1000 ppm through different time periods.

Oils	Mortality % (mean ± SD)/h					Grouping
	0.5	2	8	24	48	
*Allium sativum*	6.67 ± 0.58^aE^	22.33 ± 1.53^D^	46.67 ± 0.58^efgiC^	81.33 ± 1.53^dB^	96.67 ± 0.58^eA^	H
*Anethum graveolens*	8.33 ± 0.58^aE^	23.33 ± 1.15^D^	48.67 ± 1.15^jC^	83.67 ± 1.53^dB^	98.33 ± 0.58^eA^	H
*Argania spinosa*	5.00 ± 1.00^aE^	11.67 ± 0.58^D^	21.67 ± 1.53^bcdC^	43.33 ± 1.53^cB^	66.67 ± 1.53^dA^	L
*Boswellia serrata*	3.33 ± 0.58^aE^	15.00 ± 1.00^D^	31.67 ± 1.53^bcdeC^	70.00 ± 1.00^dB^	90.00 ± 1.00^eA^	M
*Brassica carinata*	3.33 ± 0.58^aE^	13.33 ± 0.58^D^	25.00 ± 1.00^bcdC^	45.00 ± 1.53^cB^	68.33 ± 2.08^dA^	L
*Camellia sinensis*	8.33 ± 0.58^aE^	23.33 ± 1.00^aC^	61.67 ± 1.531^jB^	100.00 ± 1.00^dA^	100.00 ± 0.58^eA^	H
*Cedrus libani*	5.00 ± 1.00^abE^	15.00 ± 0.00^aD^	25.00 ± 1.00^cC^	56.67 ± 1.00^dB^	78.33 ± 1.53^eA^	L
*Citrullus colocynthis*	3.33 ± 0.58^aE^	11.67 ± 0.58^cdeD^	33.33 ± 0.58^defgC^	65.00 ± 1.00^defB^	75.00 ± 1.00^deA^	L
*Crocus sativus*	3.33 ± 0.58^aE^	10.00 ± 1.00^defD^	21.67 ± 1.15^hijC^	39.33 ± 1.00^hiB^	62.33 ± 1.00^fgA^	L
*Cucurbita maxima*	3.33 ± 0.58^aE^	10.00 ± 1.00^defD^	21.67 ± 1.53^hijC^	48.33 ± 1.53^ghB^	65.00 ± 1.35^efgA^	L
*Cuminum cyminum*	3.33 ± 0.58^aE^	8.33 ± 0.58^efD^	33.33 ± 1.53^defgC^	63.33 ± 1.53^defB^	88.33 ± 1.53^bcA^	M
*Cupressus sempervirens*	5.00 ± 1.00^aE^	8.33 ± 0.58^efD^	16.67 ± 0.58^ijC^	41.67 ± 2.08^hiB^	63.33 ± 2.00^fgA^	L
*Curcuma aromatic*	5.00 ± 1.00^aE^	16.67 ± 1.53^abcdeD^	35.00 ± 1.73^defC^	71.67 ± 1.53^cdB^	88.33 ± 1.53^bcA^	M
*Curcuma longa*	5.00 ± 1.00^aE^	10.00 ± 1.00^defD^	20.00 ± 1.00^ijC^	40.00 ± 2.08^hiB^	61.67 ± 1.53^fgA^	L
*Foeniculum vulgare*	8.33 ± 0.58^aE^	25.00 ± 1.15^aC^	63.33 ± 0.58^aB^	100.00 ± 1.00^aA^	100.00 ± 0.00^aA^	H
*Gadus morhua*	5.00 ± 1.00^abE^	13.33 ± 0.58^bcdeD^	31.67 ± 1.53^defghC^	55.00 ± 1.00^fgB^	75.00 ± 1.00^deA^	L
*Lepidium sativum*	6.67 ± 0.58^aE^	15.00 ± 1.00^abcdeD^	36.67 ± 1.15^deC^	70.00 ± 1.00^cdeB^	90.00 ± 1.00^abcA^	M
*Linum usitatissimum*	3.33 ± 0.58^aE^	15.00 ± 1.00^abcdeD^	40.00 ± 1.00^cdC^	55.00 ± 1.00^fgB^	75.00 ± 1.00^deA^	L
*Melaleuca alternifolia*	6.67 ± 0.58^aE^	10.00 ± 1.00^defD^	40.00 ± 1.00^cdC^	71.67 ± 1.53^cdB^	81.67 ± 0.58^cdA^	M
*Nigella sativa*	5.00 ± 1.00^aE^	20.00 ± 1.00^abcdD^	50.00 ± 1.00^bcC^	78.67 ± 1.53^bcB^	95.00 ± 1.00^abA^	H
*Panax ginseng*	5.00 ± .1.00^aE^	11.67 ± 0.58^cdeD^	30.00 ± 1.73^defghC^	48.33 ± 1.53^ghB^	71.67 ± 1.15^defA^	L
*Piper nigrum*	5.00 ± 1.00^aE^	20.00 ± 1.00^abcdD^	38.33 ± 0.58^dC^	70.00 ± 1.00^cdeB^	88.33 ± 1.58^bcA^	M
*Prunus dulcis*	3.33 ± 0.57^aE^	13.33 ± 0.33^bcdeD^	31.67 ± 0.88^defghC^	50.00 ± 0.57^ghB^	75.00 ± 0.57^deA^	L
*Ruta chalepensis*	3.33 ± 0.58^aE^	15.00 ± 1.00^abcdeD^	33.33 ± 2.08^defgC^	60.00 ± 2.00^efB^	80.00 ± 1.00^cdA^	L
*Salvia officinalis*	6.67 ± 0.58^aE^	21.67 ± 1.53^abcD^	51.67 ± 1.53^bC^	80.00 ± 1.53^bcB^	97.33 ± 1.00^abA^	H
*Sesamum indicum*	3.33 ± 0.58^aE^	8.33 ± 1.15^efD^	15.00 ± 1.00^jC^	36.67 ± 1.15^iB^	60.00 ± 1.15^gA^	L
*Simmondsia chinensis*	5.00 ± 1.00^aE^	11.67 ± 0.58^cdeD^	36.67 ± 1.53^deC^	70.00 ± 2.0^cdeB^	91.67 ± 0.58^abA^	M
*Syzygium aromaticum*	5.00 ± 1.00^aE^	13.33 ± 0.58^bcdeD^	23.33 ± 1.15^ghijC^	50.00 ± 1.00^ghB^	76.673 ± 1.53^dA^	L
*Tilia americana*	5.00 ± 0.57^aE^	15.00 ± 0.0^abcdeD^	25.00 ± 0.57^fghijC^	56.67 ± 0.88^fgB^	88.33 ± 0.88^bcA^	L
*Thymus vulgaris*	8.33 ± 0.58^aE^	21.67 ± 0.58^abcD^	58.33 ± 2.08^abC^	85.00 ± 0.58^bB^	100.00 ± 1.00^aA^	H
*Viola odorata*	8.33 ± 0.58^aE^	23.33 ± 1.00^abD^	58.67 ± 1.53^abC^	89.67 ± 1.53^abB^	100.00 ± 0.00^aA^	H
*Zingiber officinale*	5.00 ± 1.00^aE^	13.33 ± 0.58^bcdeD^	26.67 ± 1.53^efghiC^	48.33 ± 1.53^ghB^	75.00 ± 1.00^deA^	L
Control	0.33 ± 0.33^aA^	0.33 ± 0.33^fA^	0.33 ± 0.33^kA^	0.33 ± 0.33^jA^	0.33 ± 0.33^hA^	L

Numbers of the same raw followed by the same small letter are not significantly different (one-way ANOVA, Duncan’s MRT, P > 0.05).

H: The highly effective (95–100% mortalities), 8 oils.

M: The moderately effective group (81–92% mortalities), 7 oils.

L.: The moderately effective group, include the rest of oils, 17 oils.

**Table 3:** Lethal time values of applied oils (1000 ppm) against *Culex pipiens* larvae.

Oil name	LT_50_ (lower–upper)	RE (LT_50_)	LT_90_ (lower–upper)	RE (LT_90_)	LT99 (lower–upper)	RE (LT_99_)	Chi (Sig)	Regrision equation
*Allium sativum*	13.95 (3.16–54.44)	2.7	31.17 (18.49–174.49)	2.2	45.20 (26.92–276.44)	**2.1**	39.30 (0.000a)	y = 0.86 + 0.06*x
*Anethum graveolens*	19.90 (11.30–36.52)	1.9	39.41 (27.22–81.32)	1.8	55.31 (37.96–120.10)	1.8	23.13 (0.000a)	y = 1.23 + 0.06*x
*Argania spinosa*	33.02 (22.75–55.92)	1.1	63.55 (45.59–120.49)	1.1	88.45 (62.33–175.00)	1.1	13.91 (0.008a)	y = 1.31 + 0.04*x
*Boswellia serrata*	20.78 (12.05–37.26)	1.8	41.01 (28.56–82.20)	1.7	57.50 (39.77–121.10)	1.7	22.42 (0.000a)	y = 1.27 + 0.06*x
*Brassica carinata*	32.09 (21.04–59.25)	1.2	62.39 (43.53–132.05)	1.1	87.09 (59.69–193.58)	1.1	17.05 (0.002a)	y = 1.33 + 0.04*x
*Camellia sinensis*	13.02 (3.56–56.12)	2.9	27.65 (16.38–172.03)	2.5	39.58 (23.51–269.84)	**2.4**	40.31 (0.000a)	y = 0.96 + 0.07*x
*Cedrus libani A*	26.87 (17.55–44.77)	1.4	52.99 (38.06–98.01)	1.3	74.29 (52.64–143.56)	1.3	16.60 (0.002a)	y = 1.24 + 0.05*x
*Citrullus colocynthis*	26.08 (12.80–65.61)	0.0	52.72 (34.03–169.10)	0.0	74.44 (47.49–257.33)	1.3	32.23 (0.000a)	y = 1.25 + 0.05*x
*Crocus sativus*	37.07 (25.39–68.56)	1.0	70.02 (49.05–147.56)	1.0	96.88 (66.53–213.77)	1.0	14.35 (0.006a)	y = 1.41 + 0.04*x
*Cucurbita maxima*	30.90 (22.00–47.60)	1.2	57.85 (43.01–97.25)	1.2	79.81 (58.44–139.44)	1.2	12.91 (0.012a)	y = 1.44 + 0.05*x
*Cuminum cyminum*	22.65 (13.54- I40.07)	1.7	43.44 (30.47–86.24)	1.6	60.39 (42.00–126.16)	1.6	22.68 (0.000a)	y = 1.39 + 0.06*x
*Cupressus sempervirens*	34.67 (26.87–47.96)	1.1	67.29 (52.45–100.54)	1.0	93.88 (71.85–144.86)	1.0	18.16 (0.66a)	y = 1.41 + 0.05*x
*Curcuma aromatic*	20.49 (10.77–39.97)	1.8	41.98 (28.40–94.24)	1.7	59.51 (40.00–141.25)	1.6	25.53 (0.000a)	y = 1.14 + 0.05*x
*Curcuma longa*	33.89 (24.46–52.94)	1.1	63.92 (47.28–109.44)	1.1	88.41 (64.29–157.09)	1.1	11.35 (0.023a)	y = 1.37 + 0.04*x
*Foeniculum vulgare*	10.22 (5.29–21.14)	3.7	20.99 (13.93–49.73)	3.3	29.77 (19.68–74.34)	3.3	21.56 (0.000a)	y = 1.06 = 0.1*x
*Gadus morhua*	27.64 (16.47–54.29)	1.4	55.69 (37.98–128.11)	1.3	78.56 (52.78–191.03)	1.2	21.54 (0.000a)	y = 1.2 + 0.04*x
*Lepidium sativum*	20.06 (11.18–36.90)	1.9	41.06 (28.31–84.97)	1.7	58.18 (39.83–126.60)	1.7	22.42 (0.000a)	y = 1.11 + 0.05*x
*Linum usitatissimum*	26.78 (12.80–77.92)	1.4	55.74 (35.22–213.81)	1.3	79.35 (49.44–328.66)	1.2	31.75 (0.000a)	y = 1.18 + 0.04*x
*Melaleuca alternifolia*	22.36 (9.11–58.90)	1.7	46.52 (29.47–159.02)	1.5	66.22 (41.73–244.98)	1.5	36.44 (0.000a)	y = 1.12 + 0.05*x
*Nigella sativa*	15.67 (5.25–46.57)	2.4	33.48 (20.57–130.64)	2.1	48.00 (29.54–202.69)	2.0	36.89 (0.000a)	y = 1.01 + 0.06*x
*Panax ginseng*	30.16 (19.05–57.39)	1.2	59.66 (41.18–131.40)	1.2	83.70 (56.80–194.15)	1.2	18.86 (0.001a)	y = 1.25 + 0.04*x
*Piper nigrum*	20.14 (9.84–41.84)	1.9	42.45 (28.17–103.75)	1.6	60.63 (40.01–157.34)	1.6	27.10 (0.000a)	y = 1.07 + 0.05*x
*Prunus dulcis*	26.75 (19.88–36.78)	2.6	58.25 (45.50–85.63)	1.4	78.56 (64.49–127.36)	1.2	21.11(0.03a)	y = 1.2 + 0.04*x
*Ruta chalepensis*	25.12 (14.06–50.27)	1.5	50.74 (34.32- 119.52)	1.4	71.63 (47.88- 178.94)	1.4	24.68 (0.000a)	y = 1.24 + 0.05
*Salvia officinalis*	15.42 (5.38–41.36)	2.4	34.12 (21.26–116.53)	2.1	49.37 (30.77–181.26)	2.0	32.84 (0.000a)	y = 0.89 + 0.06*x
*Sesamum indicum*	37.64 (32.87–44.04)	1.0	68.08 (58.97–81.70)	1.0	92.89 (79.68–112.98)	1.0	8.60 (0.720a)	y = 1.54 + 0.04*x
*Simmondsia chinensis*	19.00 (14.03–25.19)	1.9	40.45 (32.52- 55.17)	1.8	57.95 (46.08- 81.12)	1.8	4.20 (0.241a)	y = 1.23 + 0.06*x
*Syzygium aromaticum*	32.14 (21.00–44.84)	1.2	63.13 (43.91–102.50)	1.1	88.39 (60.37–19.40)	1.1	16.81 (0.031a)	y = 1.26 + 0.04*x
*Tilia americana*	26.03 (19.61–35.05)	1.4	52 (43.55–78.29)	1.3	78.62 (61.30–115.31)	1.2	16.6 (0.471a)	y = 1.24 + 0.05*x
*Thymus vulgaris*	9.67 (3.58–33.79)	3.9	21.89 (13.29–104.01)	3.2	31.86 (19.19–163.28)	**3.0**	33.04 (0.000a)	y = 0.88 + 0.09*x
*Viola odorata*	10.31 (3.88–28.58)	3.6	22.15 (13.76–78.00)	3.2	31.81 (19.76–120.35)	**3.0**	29.95 (0.000a)	y = .96 + 0.09*x
*Zingiber officinale*	29.27 (19.73–48.49)	1.3	57.30(41.31–105.43)	1.2	80.16 (56.91–153.86)	1.2	14.90 (0.005a)	y = 1.26 + 0.04*x
Reference oil	*Sesamum indicum*		*Crocus sativus*					

*RE* Relative efficacy.

Significant values are in [bold].

The efficacy of oils could be classified, 48 h post-treatment (PT) as the highly effective group (H group) inducing 95–100% mortalities, including eight oils: *Allium sativum, Anethum graveolens, Camellia sinensis, Foeniculum vulgare, Nigella sativa, Salvia officinalis, T. vulgaris,* and *Viola odorata*. *Camellia sinensis* and *F. vulgare* provided 100%, 24 h PT (Table 2).

The LT_50_ values of the H group ranged from 9.67 (*T. vulgaris*) to 19.91 (*An. graveolens*) hours and those of LT_99_ values ranged from 29.97 (*Foeniculum vulgare*) to 55.32 (*An. graveolens*). The relative effects (RE) of such oils according to LT_50_ values were 2.7, 1.9, 2.9, 3.7, 2.4, 2.4, 3.9, and 3.6 times, respectively, times than *S. indicum*; whereas those of LT_99_ values were 2.1, 1.8, 2.4, 3.3, 2.0, 2.0, 3.0, and 3.0 times, respectively, than *C. sativus*. The Chi-square, significance, and regression equations were provided for all teste oils (Table 3).

**Table 4:** Kruskal–Wallis test for larval mosquito mortality (%) of plant oil groups at 1000 ppm.

Oil groups	Mortality % (mean ± SD)*				
	0.5 h	2 h	8 h	24 h	48 h
Low	4.2 ± 0.847	12.3 ± 2.278	25.980 ± 6.590	49.4 ± 7.838	71.6 ± 7.39
Medium	5.0 ± 1.361	13.8 ± 4.050	35.950 ± 2.864	69.5 ± 2.841	88.3 ± 3.191
High	7.5 ± 1.260	22.7 ± 1.527	54.792 ± 6.389	87.1 ± 8.533	98.3 ± 1.992
Chi-Square	16.909**	18.152**	23.037**	25.391**	25.098**
df	2	2	2	2	2
Asymp. Sig	0.001	0.001	0.001	0.001	0.001

*Means produced by non-parametric analysis (Kruskal–Wallis, p 0.05).

**The *X*^*2*^ value is sig. at significant level 1%

H: The highly effective group (95–100% mortalities) are 8 oils (*A. sativum*, *A. graveolens, C. sinensis, F. vulgare, N. sativa, S. officinalis, T. vulgaris,* and *V. odorata).*

M: The moderately effective group (81–92% mortalities) are 7 oils (*B. serrata, C. cyminum, C. aromatic, L. sativum, M. alternifolia, P. nigrum,*and *S. chinensis).*

L.: The moderately effective group are included the rest of oils, 17 oils (*A. spinosa, B. carinata, C. libani, C. colocynthis, C. sativus, C. maxima, C. sempervirens, C. longa, G. morhua, L. usitatissimum, P. ginseng, P. dulcis, R. chalepensis, S. indicum, S.aromaticum, T. americana,* and *Z. officinale).*

The moderately effective (M group) group of oils resulted in 81–92% mortalities 48 h PT, including *B. serrata, C. cyminum, C. aromatic, L. sativum, M. alternifolia, P. nigrum,* and *S. chinensis.* They provided 63.33–71.67% mortalities, 24 h PT (Table 2).

The LT_50_ values of M group ranged from 19.00 (*S. chinensis*) to 22.65 (*C. cyminum*) hours and those of LT_99_ values ranged from 57.95 (*S. chinensis*) to 66.22 (*M. alternifolia*) (Table 3). Their RE regarding the LT_50_ values were 1.8, 1.7, 1.8, 1.9, 1.7, 1.9, and 1.9 times than *S. indicum*, respectively, whereas those of LT_99_ values were 1.7, 1.6, 1.6, 1.7, 1.5, 1.6, and 1.8 times than *C. sativus,* respectively (Table 3).

The least effective group (L group) included the other 17 oils, and the least effective ones were *C. sativus*, and *S. indicum,* providing 62.33 and 60.00% mortalities, 48 h PT, whereas their LT_50_ values were 37.07 and 37.64 h and their LT_99_ values were 96.88 and 92.89 h, respectively (Table 3).

Furthermore, the Kruskal–Wallis test was performed to compare the mean differences of more than two groups, followed by the Mann–Whitney test to compare the mean differences between groups. Whereas Kruskal–Wallis and Friedman's tests showed there are significant indications between the three groups at different times (*P* = 0.001) (Tables 4 and 5).

**Table 5:** Friedman test for larval mosquito mortality (%) of plant oil groups at 1000 ppm.

Oil groups	0.5 h	2 h	8 h	24 h	48 h	Chi^2^ Df = 4
Low	4.2 ± 0.847	12.3 ± 2.278	25.980 ± 6.590	49.4 ± 7.838	71.6 ± 7.39	68**
Medium	5.0 ± 1.361	13.8 ± 4.050	35.950 ± 2.864	69.5 ± 2.841	88.3 ± 3.191	28**
High	7.5 ± 1.260	22.7 ± 1.527	54.792 ± 6.389	87.1 ± 8.533	98.3 ± 1.992	31.7**
total	5.21 ± 1.733	15.21 ± 5.111	35.36 ± 13.379	63.23 ± 17.613	81.93 ± 13.09	127.6**

**The *X*^*2*^ value is sig. at significant level 1%

*Viola odorata, A. graveolens, T. vulgaris,* and *N. sativa* provide 100% adult mortalities PT with 10. 25. 20, and 25%. The mortality percentages of the adults subjected to 10% of oils (H group) were 48.89%, 88.39, 63.94, 51.54, 92.96, 44.44, 72.22, and 100.0% for *A. sativum, An. graveolens, C. sinensis, F. vulgare, N. sativa, S. officinalis T. vulgaris,* and *V. odorata,* respectively. Their adulticidal LC_50_ values, 24 h PT, were 15.57, 2.42, 9.01, 15.07, 3.42, 20.46, 3.08, and 1.88%; whereas their LC_90_ values were 38.86, 9.47, 32.18, 33.34, 5.44, 50.76, 16.08, and 7.37%, respectively. *Salvia officinalis* followed by *A. sativum* were the least effective oils against adults. According to LC_90_*, N. sativa, V. odorata* and *An. graveolens* killed mosquitoes 9.3, 6.9, and 5.4 times more than *S. officinalis* (Table 6).

**Table 6:** The adulticidal effects of selected plant oils against *Culex pipiens* after 24 h post-treatments.

Oil name	Conc. %	Mortality% (mean ± SD)	LC_50_ (lower–upper limit)	RE (LC_50_)	LC_90_ (lower–upper limit)	RE (LC_90_)	LC_95_ (lower–upper limit)	RE (LC_95_)	Chi (Sig)	Equation
*Allium sativum*	0	0 ± 0e	15.57 (8.49–28.46)	2.4	38.86 (26.79–81.87)	1.9	45.47 (31.19–97.80)	1.9	24.40 (0.000a)	Y = 0.051 + 0.008*x
	0.5	20.00 ± 6.67d								
	2.0	24.44 ± 5.88d								
	5.0	42.22 ± 2.22c								
	10	48.89 ± 4.44c								
	20	62.22 ± 8.01b								
	40	86.67 ± 3.85a								
*Anethum graveolens*	0	6.37 ± 18.75d	2.42 (0.08–4.22)	8.05	9.47 (4.66–17.80)	5.4	23.25 (7.17–129.13)	2.6	33.254 (.000a)	Y = 0.242 + 0.130*x
	0.1	36.86 ± 15.46bc								
	0.5	41.66 ± 27.57b								
	2	46.12 ± 11.77b								
	5	75.96 ± 18.84a								
	10	88.39 ± 7.27a								
	20	91.85 ± 9.24a								
	25	100.00 ± 0.00a								
*Camellia sinensis*	0	3.57 ± 20.00c	9.01 (− 17.75 to 23.09)	2.3	32.18 (19.96–170.57)	1.6	38.754 (24.052–218.98)	1.5	26.52 (0.000a)	Y = 0.644 + 0.106*x
	2	51.51 ± 2.62b								
	5	61.21 ± 6.30ab								
	10	63.94 ± 10.22ab								
	15	75.35 ± 29.22ab								
	20	78.78 ± 16.87ab								
	25	91.99 ± 0.45a								
*Foeniculum vulgare*	0	10.50 ± 25.00d	15.07 (0.10–104.60)	1.4	33.34 (21.67–789.17)	1.5	38.53 (24.63–986.39)	1.5	22.19 (0.000a)	Y = 0.331 + 0.03*x
	5	36.73 ± 16.93bc								
	10	51.54 ± 11.47ab								
	15	51.70 ± 2.27ab								
	20	59.00 ± 16.87ab								
	25	75.96 ± 1.36a								
*Nigella sativa*	0	4.95 ± 20.61e	3.42 (− 53.96 to 30.15)	6.0	5.44 (− 14.41 to 84.13)	9.3	29.95 (15.87-1184.48)	2.0	57.88 (0.000a)	Y = 0.261 + 0.06*x
	0.05	41.87 ± 12.75 cd								
	0.1	60.68 ± 3.73bc								
	0.5	72.91 ± 6.45ab								
	1	74.54 ± 19.78ab								
	2	78.09 ± 18.28ab								
	10	92.96 ± 9.44ab								
	25	100.00 ± 6.11ab								
*Salvia officinalis*	0	0 ± 0e	20.46 (11.34–45.85)	1.0	50.76 (33.24–140.52)	1.0	59.35 (38.59–168.23)	1.0	25.35 (0.000a)	Y = 0.8022 + 0.091*x
	0.5	17.78 ± 2.22d								
	2.0	22.22 ± 2.22d								
	5.0	37.78 ± 4.45c								
	10	44.44 ± 4.44bc								
	20	53.33 ± 3.85b								
	40	73.33 ± 7.70a								
*Thymus vulgaris*	0	3.57 ± 7.15c	3.08 (− 3.29 to 7.48)	6.6	16.08 (10.43–41.60)	3.2	19.76 (12.83–52.76)	3.0	34.12 (0.000a)	Y = 0.350 + 0.091*x
	0.1	38.74 ± 4.28b								
	0.5	61.66 ± 7.26ab								
	2	69.82 ± 9.85ab								
	10	72.22 ± 14.69ab								
	20	100.00 ± 0.00a								
*Viola odorata*	0	3.57 ± 7.15d	1.88 (− 1.80 to 5.29)	10.8	7.37 (4.46–29.82)	6.9	8.92 (5.43–37.58)	6.6	21.99 (0.001a)	Y = 0.190 + 0.112*x
	0.1	50.00 ± 10.00c								
	0.5	54.95 ± 15.61c								
	1	57.50 ± 19.20c								
	2	65.83 ± 13.21bc								
	6	85.05 ± 13.62ab								
	10	100.00 ± 0.00a								
Reference oils			*Salvia officinalis*							

### Oil phytochemical analysis

**Figure 1:**
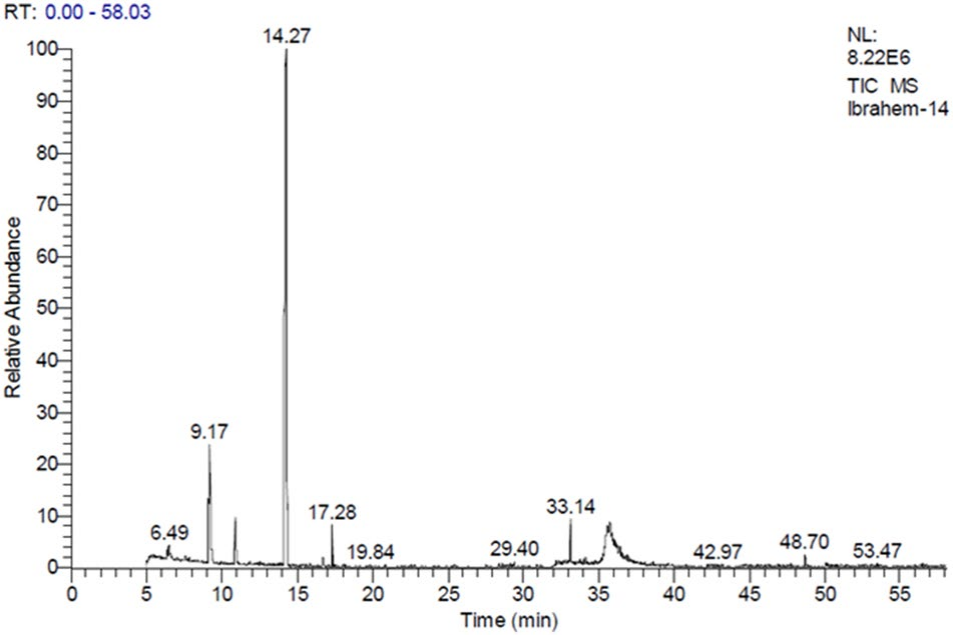
GC/MS analysis of the *Foeniculum vulgare Mill.*

Phytochemical analysis of oils of *F. vulgare Mill.*, *An. graveolens L.*, *V. odorata L.*, *T. vulgaris L.*, *A. sativum*, *S. officinalis* and *C. sinensis* by GC/MS and HPLC analysis revealed their major compounds. *F. vulgare* oil contains Estragole (70.36%); Limonene (8.96%) and 1,3,3-trimethyl Bicyclo [2.2.1]heptan-2-one (2.81%) (Table 7 and Fig. 1).

**Table 7:** GC/MS analysis of the *Foeniculum vulgare Mill.*

Peak no.	R_t_ (min.)	MW	MF	Area %	Probabilities of the detected compounds
1	5.03	40	C3H4	0.14	1-Propyne
2	5.22	138	C7H10N2O	0.26	2,3,3a,4,7,7a-Hexahydro-1H-benzimidazol-2-one
3	5.28	348	C19H22ClFN2O	1.06	1-Chloro-3-(3-fluorobenzoyl)-4-(2-(diethylamino)ethylamino)benzene
4	6.38	136	C10H16	0.41	Sabinene
5	6.49	262	C12H23O4P	1.01	Dimethyl{[2,2-dimethyl-3-(2′-methylprop-1′-cyclopropyl]methyl}phosphate
6	7.57	670	C44H27DN4Ni	0.15	(5,10,15,20-tetraphenyl[2-(2)H1]prophyrin-ato)zinx(II)
7	9.17	136	C10H16	8.96	Limonene
8	10.90	152	C10H16O	2.81	1,3,3-trimethyl Bicyclo[2.2.1]heptan-2-one
10	14.26	148	C10H12O	70.36	Estragole
11	14.72	818	C44H28Br2N4Ti	0.11	Tetraphenylporphyrinatodibromotitanium (IV)
12	16.70	166	C11H18O	0.47	3,7-Dimethyl-2,6-Nonadienal
13	17.28	152	C10H16O	1.41	2,4-Decadienal
14	18.07	194	C14H26	0.17	1,1′-Bicycloheptyl
15	29.40	300	C17H36O2Si	0.20	Tetradecanoic acid, trimethylsilyl ester
16	32.19	160	C10H21F	0.15	Fluoro decane
17	32.36	244	C13H24O4	0.11	Oxalic acid isohexylpentyl ester
18	33.14	328	C19H40O2Si	1.74	Hexadecanoic acid, trimethylsilyl ester
19	33.78	282	C18H34O2	0.15	(Z) 9-Octadecenoic acid
20	34.03	138	C10H18	0.25	7-Methyl-1-nonyne
21	34.12	282	C18H34O2	0.30	(Z) 9-Octadecenoic acid
22	34.58	256	C16H32O2	0.12	Hexadecanoic acid
23	35.57	280	C18H32O2	1.44	(Z,Z) 9,12-Octadecadienoic acid
24	35.64	280	C18H32O2	1.03	(Z,Z) 9,12-Octadecadienoic acid
25	35.70	356	C21H40O4	0.53	2,3-Dihydroxypropylelaidate
26	35.76	238	C16H30O	1.67	Z-7-Hexadecenal
27	36.25	280	C18H32O2	0.23	(Z,Z )9,12-Octadecadienoic acid
28	36.38	266	C18H34O	0.43	12-Octadecenal
29	42.83	142	C9H18O	0.13	Nonanal
31	46.93	660	C20Cl12	0.13	Dodecachloroperylene
32	48.70	295	C20H25NO	0.61	(R)-1-[N-1-cyclopentylpropionylamino-1-ethyl]naphthalene
33	50.05	354	C20H18O6	0.38	Isosesamin

**Figure 2:**
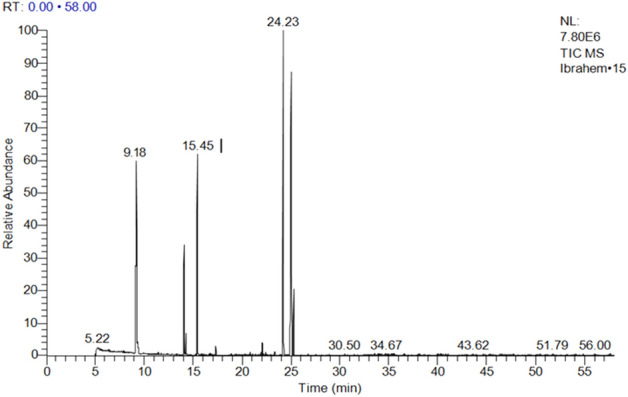
GC/MS analysis of the *Anethum graveolens L.*

*Anethum graveolens* showed abundance of 4-Pyridinecarbaldehyde-4-propyl-3-thiosemicarbazone (32.13%); 1,5-dimethyl-1,5-Cyclooctadiene (17.19%); Dihydrocarvone (5.98%); 3a(1H)-Azulenol,2,3,4,5,8,8a-hexahydro-6,8-adimethyl-3-(1-methylethyl),[3R-(3à,3aà,8aà)] (Carotol) (21.26%); and tricyclic compound Daucol (2.39%) (Table 8 and Fig. 2).

**Table 8:** GC/MS analysis of the *Anethum graveolens L.*

Peak no.	R_t_ (min.)	MW	MF	Area %	Probabilities of the detected compounds
1	5.14	238	C13H18O4	0.49	Diethyl 3,4-bis(methylene)cyclopentane-1,1-dicarboxylate
2	5.21	600	C33H28O11	0.69	(2′S,3S,3′S,P)-hydroxyanhydrophlegmacin-9,10-quinone 8′-O-methylether
3	7.65	290	C19H30O2	0.06	2-(2′-Isopropenyldec-2′-enyl)methylcyclopentane-1,3-dione
4	9.18	136	C10H16	17.19	1,5-Dimethyl-1,5-Cyclooctadiene
5	9.35	136	C10H16	0.23	dl-Limonene
6	14.05	152	C10H16O	5.98	Dihydrocarvone
7	14.25	152	C10H16O	0.86	CIS-DIHYDROCARVONE
8	15.44	150	C10H14O	14.62	2-Methyl-5-(1-methylethenyl)2-Cyclohexen-1-one
9	15.80	733	C44H28Cl2N4V	0.07	Dichloro(5,10,15,20-tetra phenylporphyrinato)vanadium
10	16.71	692	C41H33FeO5P	0.13	Dicarbonyl(1,3-5-ü-6-phenyl-2-(phenylethynyl)cyclohept-4-ene-1,3-diyl) triphenoxyphosphaneiron
11	17.29	110	C8H14	0.47	octahydro Pentalene
12	18.89	675	C44H28CuN4	0.09	(5,10,15,20-tetraphenyl[2-(2)H1]prophyrinato)copper(II)
13	20.82	204	C15H24	0.10	à-Humulene
14	21.36	686	C37H24Cl2N6O4	0.08	2,2-Bis[4[[4-chloro-6-(3-ethynylphenoxy)-1,3,5-triazin-2-yl]oxy]phenyl]propane
15	21.92	134	C10H14	0.14	1,2,3,4-Tetramethyl-5-methylenecyclopenta-1,3-diene
16	22.07	204	C15H24	0.38	á –Bisabolene
17	22.16	648	C35H38Cl2N4O4	0.11	2,4-bis(á-chloroethyl)-6,7-bis[á-methoxycarbonylethyl]-1,3,5-trimethylporphyrin
18	22.36	640	C32H64O5Si4	0.23	OTETRAKIS(TRIMETHYLSILYL)3,5-DIHYDROXY-2-(3-HYDROXY-1-OCTENYL)CYCLOPENTANEHEPTANOATE
19	23.34	208	C14H24O	0.18	3-Oxabicyclo[3.3.1]non-6-ene
20	24.23	222	C15H26O	21.26	3a(1H)-Azulenol,2,3,4,5,8,8a-hexahydro-6,8-adimethyl-3-(1-methylethyl),[3R-(3à,3aà,8aà)]
21	24.57	572	C23H26Br2O7	0.10	Dibromogomisin A
22	25.05	222	C10H14N4S	32.13	4-Pyridinecarbaldehyde-4-propyl-3-thiosemicarbazone
23	25.28	238	C15H26O2	2.39	Daucol
24	26.01	194	C12H18O2	0.06	3-(1-Hydroxyhexyl)phenol
25	27.54	220	C15H24O	0.06	Trans-Z-à-Bisaboleneepoxide
26	33.01	2598	N/A	0.07	YGRKKRRQRRRGPVKRRLDL/5
27	34.16	691	C51H33NO2	0.07	2,6-Bis(2,3,5-triphenyl-4-oxocyclopentadienyl)pyridine
28	35.47	733	C44H28Cl2N4V	0.08	Dichloro(5,10,15,20-tetraphenylporphyrinato)vanadium
29	40.31	739	C39H81NO4Si4	0.13	(3S,4R,1′E,2″R,3″R)-1-tertButyldimethylsilyl-4-(3′-tertbutyldimethylsilyloxy-2′-methylprop-1′-enyl)-3-(1″,3″ di(tertbutyldimethylsilyloxy)-2″-methylhex-5″-yl]-3-methylazetidin-2-one
31	43.48	114	C6H10O2	0.13	3,4-Hexanedione
32	50.56	680	C35H40O5Si5	0.06	Pentamethylpentaphenylcyclopentasiloxane
33	51.11	733	C44H28Cl2N4V	0.09	Dichloro(5,10,15,20-tetraphenylporphyrinato)vanadium

**Figure 3:**
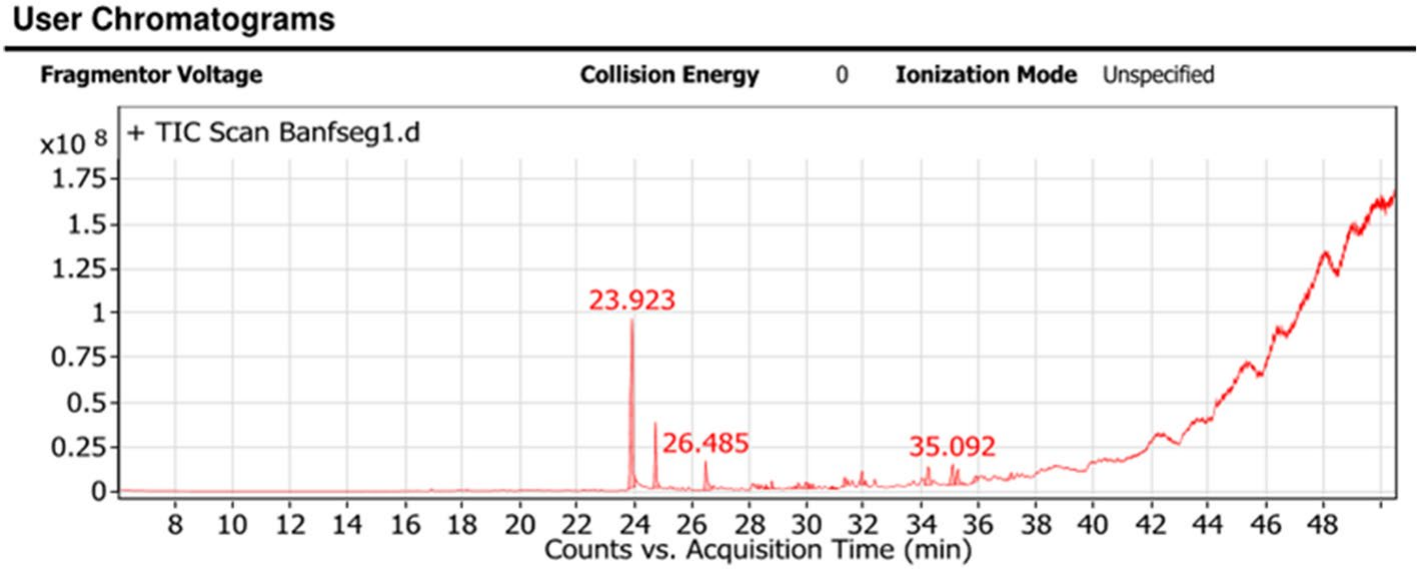
GC/MS analysis of the sample *Viola odorata L.*

*Viola odorata L.* oil contains Diphenyl ether (42.04%); alpha.-Ionone(11.87%); (Z)-5-(4-tert-Butyl-1-hydroxycyclohexyl)-3-methylpent-2-en-4-yne (7.22%); 2,3,3a,4,5,5a,6,7,9a,9b-decahydro-3,5a,9-trimethyl-7,9a-peroxy Naphtho-[1,2-b]furan-2-one (6.6%); 2-hexyl-1-Decanol (4.15%); and hexadecahydro-Pyrene (2.79%) (Table 9 and Fig. 3).

**Table 9:** GC/MS analysis of the *Viola odorata L.*

Peak no.	R_t_ (min.)	MW	MF	Area %	Probabilities of the detected compounds
1	23.923	170	C12H10O	42.04	Diphenyl ether
2	24.735	192	C13H20O	11.87	.alpha.-Ionone
3	26.485	192	C13H20O	7.73	3-Buten-2-one, 4-(2,6,6-trimethyl-1-cyclohexen-1-yl)
4	28.317	236	C15H24O2	0.61	Limonen-6-ol, pivalate
5	28.58	226	C13H22O3	0.9	2-Hydroxy-1,1,10-trimethyl-6,9-epidioxydecalin
6	28.786	238	C16H30O	1.26	7-Hexadecenal, (Z)-
7	29.599	236	C16H28O	0.83	7,11-Hexadecadienal
8	29.713	296	C20H40O	1.48	Phytol
9	29.959	242	C16H34O	2.15	2-Hexyl-1-Decanol
10	30.074	378	C25H46O2	1.09	Undec-10-ynoic acid, tetradecyl ester
11	30.211	296	C20H40O	1.02	PHYTOL ISOMER
12	30.881	266	C16H26O3	0.67	2-Dodecen-1-yl(-)succinic anhydride
13	31.338	242	C16H34O	2.14	1-Decanol, 2-hexyl-
14	31.939	218	C16H26	2.79	hexadecahydroPyrene
15	32.054	240	C17H36	0.7	Tetradecane, 2,6,10-trimethyl
16	34.245	250	C16H26O2	7.22	(Z)-5-(4-tert-Butyl-1-hydroxycyclohexyl)-3-methylpent-2-en-4-yne
17	35.092	264	C15H20O4	6.6	2,3,3a,4,5,5a,6,7,9a,9b-decahydro-3,5a,9-trimethyl-7,9a-peroxy Naphtho[1,2-b]furan-2-one
18	35.269	264	C15H20O4	4.73	2,3,3a,4,5,5a,6,7,9a,9b-decahydro-3,5a,9-trimethyl-7,9a-peroxy Naphtho [1,2-b]furan-2-one
19	35.905	242	C16H34O	2.19	2-hexyl-1-Decanol
20	37.146	266	C18H34O	1.89	Z,E-2,13-Octadecadien-1-ol
21	23.923	170	C12H10O	0.78	Diphenyl ether

**Figure 4:**
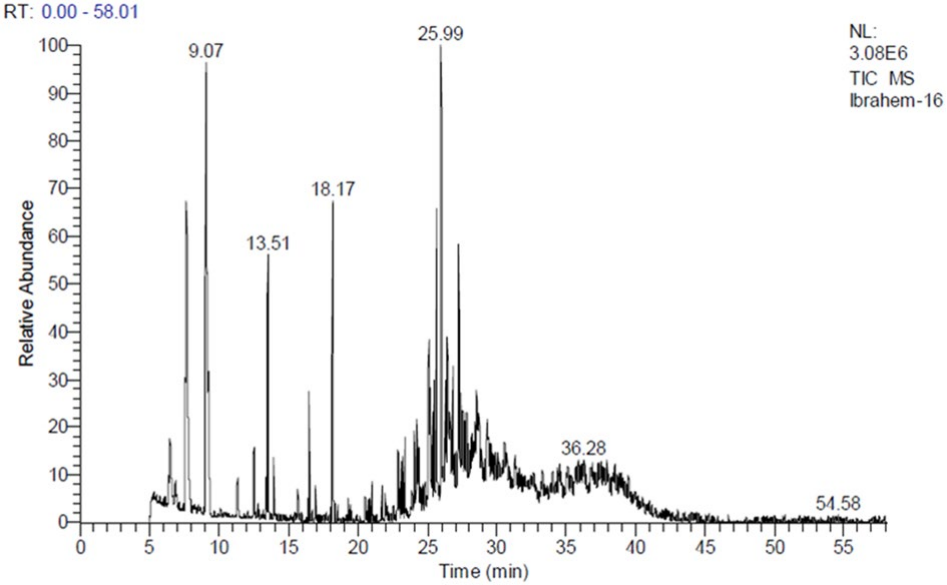
GC/MS analysis of *Thymus vulgaris L.*

*Thymus vulgaris* oil included 2-Ethynyl-3-hydroxypyridine (12.37%); 2-á-pinene(8.92%),2,5-Dipropoxybenzalde-hyde (7.70%); 5-Amino-8-cyano-7-methoxy-3,4-dihydro-3-methy-l1,6-naphthyridin- (1H)-one (5.05%); à-terpinyl acetate (5.00%); 4-methyl-1-(1-methyl-ethyl)-3-Cyclohexen-1-ol (4.73%), 3-(6,6-Dimethyl-5-oxohept-2-enyl)-cyclo-heptanone (4.54%); 10-Methylnonadecane(4.12%); 9-methyl Nonadecane-(3.55%); n1,1′-oxybis Decane (2.36%); 7,11-Hexadecadienal (2.14%); and (2R,3R)-3- (2-Methoxy-4-methylphenyl)-2,3-dimethylcyclopentanone (2.01%) (Table 10 and Fig. 4).

**Table 10:** GC/MS analysis of *Thymus vulgaris L.*

Peak no.	R_t_ (min.)	MW	MF	Area %	Probabilities of the detected compounds
1	5.1	208	C13H20O2	0.86	TRANS-á-IONON-5,6-EPOXIDE
2	5.23	122	C8H15B	0.79	1-Borabicyclo[4.3.0]nonane
3	6.46	136	C10H16	1.85	Tricyclene
4	6.86	136	C10H16	0.69	Camphene
5	7.64	136	C10H16	8.92	2-á-pinene
6	9.07	119	C7H5NO	12.37	2-Ethynyl-3-hydroxypyridine
7	11.32	196	C12H20O2	0.68	Linalyl acetate
8	12.50	152	C10H16O	1.27	(1S) Bicyclo[2.2.1]heptan-2-one, 1,7,7-trimethyl
9	13.39	156	C10H20O	0.78	1-Methyl-4-(1-methylethyl)Cyclohexanol
10	13.51	154	C10H18O	4.73	4-Methyl-1-(1-methylethyl)-3-Cyclohexen-1-ol
11	13.91	154	C10H18O	1.13	à,à,4-trimethyl (S) 3-Cyclohexene-1-methanol
12	15.67	182	C11H18O2	0.63	linalyl formate
13	16.48	196	C12H20O2	1.76	EXOBORNYL ACETATE
14	18.17	196	C12H20O2	5.00	à-terpinyl acetate
15	20.52	142	C9H18O	0.56	3-Ethylheptanal
16	21.94	268	C19H40	0.58	Nonadecane
17	22.84	199	C9H13NO4	1.87	*2S,7S* Methyl-2-Hydroxy-3-oxotetrahydro-1-Hpyrrolizine-7a-(*5H*)-carboxylate
18	22.97	226	C16H34	0.92	Pentadecane-5-methyl
19	23.10	212	C15H32	0.75	3-ethyl Tridecane
20	23.22	348	C19H40O3S	0.84	hexyltridecyl ester Sulfurous acid
21	23.39	226	C16H34	1.09	3-methyl Pentadecane
22	24.06	168	C8H12N2O2	1.52	1,6-diisocyanato Hexane
23	24.24	298	C20H42O	2.36	1,1′-oxybis Decane,
24	24.40	282	C20H42	0.81	Eicosane
25	24.65	334	C18H38O3S	0.57	Sulfurous acid, butyltetradecyl ester
26	25.10	282	C20H42	4.12	10-Methylnonadecane
27	25.24	268	C19H40	1.00	7-hexyl Tridecane
28	25.37	334	C18H38O3S	1.10	6-Tetradecanesulfonic acid, butyl ester
29	25.49	334	C18H38O3S	1.44	6-Tetradecanesulfonic acid, butyl ester
31	25.68	250	C16H26O2	4.54	3-(6,6-Dimethyl-5-oxohept-2-enyl)-cycloheptanone
32	25.98	222	C13H18O3	7.70	2,5-Dipropoxybenzaldehyde
33	26.30	352	C25H52	1.33	Pentacosane
34	26.44	282	C20H42	3.55	9-methyl, Nonadecane
35	26.62	224	C16H32	1.08	1-Hexadecene
36	26.84	236	C16H28O	2.14	7,11-Hexadecadienal
37	27.25	232	C11H12N4O2	5.05	5-Amino-8-cyano-7-methoxy-3,4-dihydro-3-methy-l1,6-naphthyridin-2(1H)-one
38	27.32	232	C15H20O2	2.01	(2R,3R)-3-(2-Methoxy-4-methylphenyl)-2,3-dimethylcyclopentanone
39	27.42	282	C20H42	0.87	2,6-dimethyl Octadecane
40	27.54	310	C22H46	0.77	8-heptyl Pentadecane
41	27.65	376	C21H44O3S	0.61	Sulfurous acid, hexyl pentadecyl ester
42	27.82	226	C16H34	0.88	Hexadecane
43	28.42	164	C5H9BrO	0.62	1-Bromo-2-methyl-3-Buten-2-ol
44	28.54	242	C16H34O	1.25	2-Hexyl-1-decanol
45	28.69	111	C7H13N	1.08	1-isocyano Hexane
46	29.32	116	C7H16O	1.94	2-ethyl 1-Pentanol
47	30.70	200	C13H28O	0.82	2-Propyldecan-1-ol
48	31.33	197	C11H19NO2	0.98	2-Ethylhexyl cyanoacetate
49	33.27	592	C41H84O	0.70	1-Hentetracontanol
50	36.28	324	C23H48	0.57	9-hexyl Heptadecane
51	37.92	366	C26H54	0.58	5,14-dibutyl Octadecane

**Figure 5:**
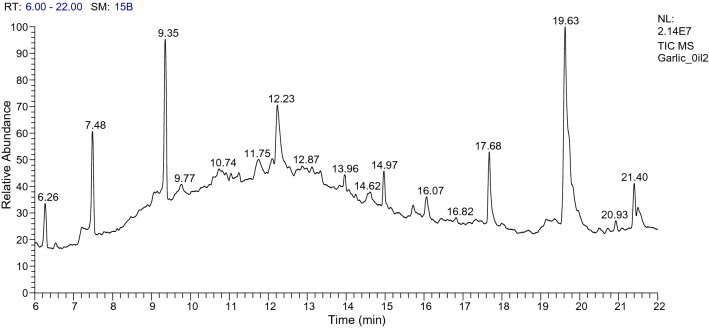
GC/MS analysis of *Allium sativum.*

*Allium sativum* contains many effective chemical compounds including the 9-Octadecenamide, (Z)-(29.07%), Trisulfide, di-2-propenyl (14.86%), and isochiapin B%2 < (8.63%) compounds (Table 11 and Fig. 5).

**Table 11:** GC/MS analysis of the *Allium sativum.*

Peak no.	R_t_ (min.)	MW	MF	Area %	Probabilities of the detected compounds
1	6.27	146	C6H10S2	4.54	Diallyl disulphide
2	7.49	152	C4H8S3	9.68	Trisulfide, methyl 2-propenyl
3	9.35	178	C6H10S3	14.86	Trisulfide, di-2-propenyl
4	12.22	350	C19H26O6	8.63	ISOCHIAPIN B %2 <
5	14.97	334	C20H30O4	3.54	1,2-Benzenedicarboxylic acid, butyl octyl ester
6	16.05	346	C19H22O6	3.11	ISOCHIAPIN B
7	17.67	387	C17H37N7O3	7.84	9-OCTADECENAMIDE
8	19.61	281	C18H35NO	29.07	9-Octadecenamide, (Z)-
10	21.40	208	C11H12O2S	4.25	3-(Benzylthio)acrylic acid, methyl ester
11	23.27	300	C19H24O3	5.86	3,17-DIOXO-11-à-HYDROXYANDROSTANE-1,4-DIENE
12	23.54	436	C26H44O5	1.82	3 Ethyl iso-allocholate
13	23.62	490	C34H50O2	6.81	CHOLEST-5-EN-3-YL BENZOATE

9-Octadecenamide, (Z)- (29.07), Trisulfide, di-2-propenyl (14.86), and ISOCHIAPIN B %2 < (8.63).

**Figure 6:**
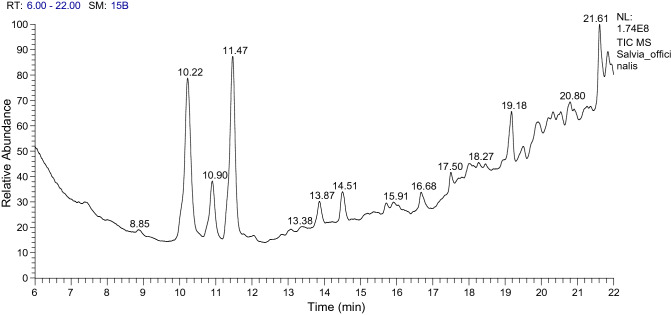
GC/MS analysis of *Salvia officinalis.*

**Figure 7:**
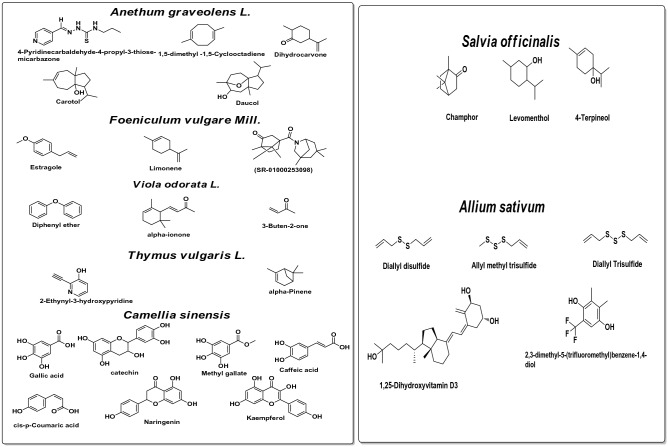
Essential oils and their most active ingredients.

*Salvia officinalis* oil showed abundance of Terpinen-4-ol (17.35%), Camphor (16.08%), 14-á-H-PREGNA (9.25%), and 1-CHLOROOCTADECANE (6.82%), (Table 12 and Fig. 6). Finally, *C. sinensis* oil is dissolved in distilled water and its major components include Gallic acid (1674 µg/ml), Catechin (421 µg/ml), Methyl gallate (1076 µg/ml), Coffeic acid (678 µg/ml), Coumaric acid (566 µg/ml), Naringenin (178 µg/ml), and Kaempferol (218 µg/ml), Table 13. Essential oils and the most active ingredients of the analyzed oils were drawn (Fig. 7).

**Table 12:** GC/MS analysis of the *Salvia officinalis.*

Peak no.	R_t_ (min.)	MW	MF	Area %	Probabilities of the detected compounds
1	10.22	152	C10H16O	16.08	Camphor
2	10.90	156	C10H20O	5.24	Cyclohexanol, 1-methyl-4-(1-methylethyl)-
3	11.47	154	C10H18O	17.35	Terpinen-4-ol
4	13.86	254	C13H24O2	2.47	Tridecanedial
5	14.50	280	C18H32O2	3.43	17-Octadecynoic acid
6	15.70	400	C28H48O	0.90	Cholestan-3-ol, 2-methylene-, (3á,5à)-
7	16.68	268	C17H32O2	1.80	7-Methyl-Z-tetradecen-1-ol acetate
8	17.50	280	C19H36O	1.63	12-Methyl-E,E-2,13-octadecadien-1-ol
10	17.99	288	C21H36	2.03	14-á-H-PREGNA
11	19.18	288	C18H37Cl	5.13	1-CHLOROOCTADECANE
12	19.51	288	C21H36	1.77	14-á-H-PREGNA
13	19.86	450	C32H66	4.33	DOTRIACONTANE
14	20.18	536	C37H76O	1.41	1-Heptatriacotanol
15	20.32	268	C16H28O3	1.15	Z-(13,14-Epoxy)tetradec-11-en-1-ol acetate
16	20.55	258	C16H34S	1.58	tert-Hexadecanethiol
17	20.80	312	C20H40O2	3.17	Ethanol, 2-(9-octadecenyloxy)-, (Z)-
18	20.90	288	C21H36	2.18	14-á-H-PREGNA
19	21.26	350	C19H26O6	0.73	ISOCHIAPIN B %2<
20	21.61	288	C18H37Cl	6.82	1-CHLOROOCTADECANE
21	21.84	294	C21H36	3.7	14-á-H-PREGNA
22	22.39	288	C21H36	0.82	1-Heptatriacotanol
23	22.47	346	C19H22O6	2.74	ISOCHIAPIN B
24	22.73	288	C21H36	9.25	14-á-H-PREGNA
25	23.09	280	C19H36O	2.20	12-Methyl-E,E-2,13-octadecadien-1-ol
26	23.23	350	C19H26O6	2.05	ISOCHIAPIN B %2 <

**Table 13:** HPLC analysis for *Camellia sinensis.*

Standard			Sample green tea		
St. compound	Conc. (µg/ml)	Area	Compound	Area	Conc. (µg/ml = µg/g)
allic acid	16.8	179.72	Gallic acid	895.77	1674.71
Chlorogenic acid	28	335.23	Chlorogenic acid	75.30	125.79
Catechin	67.5	584.16	Catechin	182.42	421.56
Methyl gallate	10.2	789.05	Methyl gallate	4163.86	1076.52
Coffeic acid	18	469.51	Coffeic acid	895.98	687.01
Syringic acid	17.2	389.86	Syringic acid	30.41	26.83
Pyro catechol	29.2	451.95	Pyro catechol	0.00	0.00
Rutin	61	457.55	Rutin	71.83	191.53
Ellagic acid	34.3	495.60	Ellagic acid	37.52	51.93
Coumaric acid	13.2	729.56	Coumaric acid	1566.70	566.93
Vanillin	12.9	543.81	Vanillin	0.00	0.00
Ferulic acid	12.4	353.45	Ferulic acid	71.09	49.88
Naringenin	15	266.56	Naringenin	158.25	178.11
Taxifolin	13.2	189.35	Taxifolin	16.08	22.42
Cinnamic acid	5.8	573.08	Cinnamic acid	0.00	0.00
Kaempferol	12	289.35	Kaempferol	263.99	218.97

## Discussion

EOs could serve as suitable alternatives to synthetic insecticides because they are relatively safe, available, and biodegradable^15^. In this study, 32 oils were evaluated against *Cx. pipiens*. *Thymus vulgare* and *C. sinensis* were the most effective larvicides (100% mortality 24 h PT). The larvicidal effect of the H group could be arranged according to their LT_50_ values (h) as follows: *T. vulgaris* (9.67), *F. vulgare* (10.22), *V. odorata* (10.31), *C. sinensis (*13.02)*, A. sativum (*13.95)*, S. officinalis (*15.42)*, N. sativa* (15.67)*,* then *An. graveolens* (19.90). On the other hand, their LT_99_ values ranged from 29.77 (*F. vulgare*) to 55.31 (*An. graveolens*).

In this study, the most effective oils against adults were *An. graveolens* and *V. odorata* followed by *T. vulgaris* then *N. sativa*. The data revealed that *F. vulgare* is a highly potent larvicide. Similarly, its oil controlled *Anopheles atroparvus*, *Culex quinquefasciatus*^23,24^*,* and *Aedes aegypti*^25^. Despite its effectiveness as larvicide in this study, *F. vulgare* was the least effective adulticide. In contrast, it induced adulticidal properties against *Cx. quinquefasciatus*^23^.

Our data indicated that *C. sinensis* was a highly effective larvicide and the less effective adulticide. Comparatively, the chemical extracts of *C. sinensis* induced larvicidal and adult repellent effects against *Cx. pipiens* providing the highest protection (100%) from the bites of starved females at the dose of 6 mg/cm^2^^26^. Moreover, its leaf extract showed larvicidal effect against *Anopheles arabiensis* and *Anopheles gambiae* (*s.s*.)^27^.

*Thymus vulgaris*d *An. graveolens* showed potent larvicidal and adulticidal effects in this work. Likewise, *T. vulgaris* has both effects against *Cx. quinquefasciatus*^28^ and *Ae. aegypti*^29^. *Thymus vulgaris* exhibited larvicidal properties, 100% mortality, against *Cx. pipiens* larvae, at 200 ppm, whereas the LC_25_ and LC_50_ vlalues indicated no effect on AChE activity, activation of the detoxification system, as indicated by an increase in GST activity and a decrease in GSH rate^30^.

Our findings agree with another study found that the most potent EOs out of 53 oils against larvae were *F. vulgare*, *T. vulgaris*, *Citrus medica* (lime), and *C. sinensis* (LC_50_ = 27.5, 31.6, 51.3, 53.5 ppm, respectively). *C. sinensis* was the most efficient EOs enhancing the efficacy of deltamethrin, co-toxic factor = 316.67, over than PBO, the positive control, co-toxic factor = 283.35)^31^.

Some oils applied in this study showed a similar larvicidal effect against *Cx. pipiens* as *N. sativa*^32,33^ and *S. officinalis*^34^. Some essential oils such as *T. vulgaris*, *S. officinalis, C. sempervirens* and *A. graveolens* had a larvicidal effect against mosquito larvae and their LC_90_ values were < 200–300 ppm. This result may be due to several reasons, including the percentages of their principal components compositions that are manipulated according to the origin of plant oil, quality of oil, susceptibility of the strain used, oil storage conditions, and technical conditions^35–37^.

Likewise our findings*, An. graveolens* and *F. vulgare* act as larvicidal, pupicidal, and oviposition deterrent agents against *M. domestica*^38^. Moreover,* Ocimum basilicum* was the most effective extract tested on *Cx. pipiens* larvae and adults^39,40^.

*Allium sativum* showed high potency against larvae in this study. A similar finding was recorded for *Cx. pipiens and Culex restuans* (LC_50_ = 7.5 and 2.7 ppm, respectively)^41^. *Argania spinosa* oil showed a low larvicidal effect in this study. A similar effect was recorded against *Cx. quinquefasciatus* larvae^42^.

*Curcuma* species was less effective in this study, but its 27 components as curcuminoids and monocarbonyl curcumin derivatives were effective larvicidal agents against *Cx. Pipiens* and *Ae. albopictus*^43^ and hexane extraction of *Curcuma longa* showed 100% larvicidal activity against *Cx. pipiens* and *Aedes albopictus* at 1000 ppm after being treated 24 h^44^.

*Zingiber officinale* and *Syzygium aromaticum* were less effective. In contrast, they were effective against *Cx. pipiens* (LC_50_ = as 71.85 and 30.75, respectively)^45^.

*Sesamum indicum* is one of the L group in this study. In contrast, petroleum ether extract showed larvidcidal, antifeedant and repellent action against *Cx. pipiens*^33^. Furthermore, EOs of *N. sativa*, *Allium cepa,* and *S. indicum*, induced larvicidal effect and their LC_50_ values against both field and laboratory strains of *Cx. pipiens* were 247.99 and 108.63; 32.11 and 2.87; and finally, 673.22 and 143.87 ppm, respectively. They influenced the pupation and adult emergence rates besides developmental abnormalities at sublethal concentrations^46^.

*Boswellia serrata* (M group) and *Brassica carinata* (L group) showed relative larvicide against *Cx. pipiens* in this study. A similar result was reported^47,48^. The lethal concentration values of Fenugreek (*Trigonella foenum-grecum*), earth almond (*Cyperus esculentus*), mustard (*Brassica compestris*), olibanum (*Boswellia serrata*), rocket (*Eruca sativa*), and parsley (*Carum ptroselinum*) were 32.42, 47.17, 71.37, and 83.36, 86.06, and 152.94 ppm, respectively. Against *Cx. pipiens* larvae. Furthermore, increasing concentrations were directly proportional to the reduction of both pupation and adult emergences rates^48^.

Some oil-resins as *Commiphora molmol, Araucaria heterophylla, Eucalyptus camaldulensis, Pistacia lentiscus,* and *Boswellia sacra* showed larvicidal activity against *Cx pipiens* larvae. The larvicidal effect 24 and 48 h PT, respectively, were for acetone extracts, 1500 ppm, of *C. molmol* (83.3% and 100% and LC_50_ = 623.52 and 300.63 ppm) and *A. heterophylla* (75% and 95% and LC_50_ = 826.03 and 384.71 ppm). On the other hand, the aqueous extract of *A. heterophylla* induced higher moralities (LC_50_ = 2819.85 ppm and 1652.50 ppm), followed by *C. molmol*, (LC_50_ = 3178.22 and 2322.53 ppm)^49^.

A similar larvicidal effect was recorded for *Rosmarinus officinalis*, hexane extract (80 and 160 ppm), reduced 100% mortality against 3rd and 4th instars larvae of *Cx. pipiens* and the toxicity increased in the pupal and adult stages^50^.

Out of 36 essential oils, red moor besom leaf oil has strong fumigation activity against *Cx. pipiens* pallens adults^51^. Similar to the adulticidal effect of the applied oils in this work, some other oils have adulticidal activities against mosquitoes as *Cedrus deodara*, *Eucalyptus citriodora*, *Cymbopogon flexuous*, *Cymbopogon* *winterianus*, *Pinus* *roxburghii*, *S. aromaticum*, and *Tagetes minuta*^52^. The Leaf Oils of *Cinnamomum* species had adulticidal activities against *Ae. aegypti* and *Aedes albopictus*^53^. EOs have adulticidal effects against *Musca domestica*^54^ as *A. sativum, S. aromaticum,* and *F. vulgare*^55^. Essential oils of *Melaleuca leucadendron* (L.) and *Callistemon citrinus* (Curtis) showed 100% adult mortality against *Aedes aegypti* (L.) and *Cx. quinquefasciatus* (Say), 24 h exposure^56^.

The results showed that *A. sativum*, and *S. officinalis* oils were effective against mosquito larvae, maybe due to the presence of a number of active secondary compounds such as ISOCHIAPIN B%2 < (sesquiterpene lactone) and 9-Octadecenamide, (Z)-that are anti-inflammatory activity^57^, also, Terpinen-4-ol and Camphor in Sage oil that these are excellent natural insecticide^58^, but these oils garlic and Sage did not show the required efficacy against adult mosquitoes.

The phytochemical analysis of this study revealed the major activated compounds of the analyzed oils. Green tea oil is a highly effective larvicide in this study contains a high amount of polyphenols that have antioxidant activity. A similar finding was reported^59^. Our data indicated that green tea oil also contains polyphenols as Gallic acid, Catechin, Methyl gallate, Coffeic acid, Coumaric acid, Naringenin, and Kaempferol which might aid in its insecticidal effect.

This study indicated that *F. vulgare* contains Estragole (70.36%) and Limonene (8.96%). Similarly, Limonene as a cyclic monoterpene has a viable insecticidal effect^60^. Besides, Estragole induced toxicity to adult fruit flies, *Ceratitis capitata*^61^. Moreover, *An. graveolens* contains thiosemicarbazone (32.13%) in this study. Likewise, thiosemicarbazide is a major component *An. graveolens* with insecticidal effect^62^. Also, Dauco and carotol are essential oils documented for *An. graveolens* in this work have repellent activity against adult *Ae. aegypti, Ae. albopictus,* and *Anopheles quadrimaculatus* Say^63^. Furthermore, *V. odorata* in the present analysis contains alpha-ionone, which revealed anti-inflammatory and analgesic effects^64^. *Thymus vulgaris* showed good a*lpha*-*pinene* and pyridine derivatives that play an important role as larvicidal and adulticidal effects against *Ae. aegypti* and growth regulator, respectively^65,66^. In addition, the combination of all constituents may promote their individual larvicidal and adulticidal effects.

The biochemical compositions showed that *T. vulgaris* oil affected the energy reserves with a marked effect on proteins and lipids^30^. The differences between our findings and those of the others could be attributed to the biological activities and the chemical composition for EOs, which could vary between plant age, tissues, geographical origin, the part used in the distillation process, distillation type, and the species. Therefore, types and levels of active constituents in each oil may be responsible for the variability in their potential against pests^16^.

## Conclusions

Diseases transmitted by mosquitoes represent global concerns. Our findings demonstrate the potential of *F. vulgare* and *C. sinensis* as the most potent larvicides and *N. sativa, V. odorata,* and *An. graveolens* as the most effective adulticides as they contain good command of different essential oils. EOs could be used for integrated mosquito control programs as larvicides or synergists for enhancing the efficacy of current adulticides^31^. Further studies are needed to develop nanoformulations that improve the efficacy and minimize applications after revealing their ecotoxicological side views.
